# An Alternative Radiation Shielding Material Based on Barium-Sulphate (BaSO_4_)-Modified Fly Ash Geopolymers

**DOI:** 10.3390/gels8040227

**Published:** 2022-04-07

**Authors:** Ammar A. Oglat, Sabri M. Shalbi

**Affiliations:** 1 Department of Medical Imaging, Faculty of Applied Medical Sciences, The Hashemite University, Zarqa 13133, Jordan; 2 Higher Institute Sciences Medical, Elkhomes 434, Libya; sabri00218@yahoo.com

**Keywords:** fly ash geopolymers (FAGP), barium sulphate (BaSO_4_), radiation shielding material, radiation dosage

## Abstract

Geopolymers are a new environmentally friendly cementitious material, and the application of geopolymers can reduce the carbon dioxide emissions caused by the development of the cement industry. Purpose: This study investigates the radiation shielding capacity of fly ash geopolymers (FAGP) as a viable alternative to conventionally used ordinary Portland cement (OPC) due to the high demand for an environmentally friendly, cost-effective and non-toxic shield material. Methods: The FAGP material was fabricated and combined with Barium sulphate (BaSO_4_) at different ratios (0, 5, 10 and 15%). Different thicknesses (3, 6 and 9 cm) of the samples were also prepared. An energy-dispersive X-ray (EDX) was used to determine the elemental percentages of the materials, which were then used to calculate their effective atomic number (Z_eff_). An ion chamber was used to detect the dose of radiation transmitted through the samples. Results: The lowest radiation dosage (34.68 µGy) and highest Z_eff_ were achieved with FAGP combined with 15% BaSO_4_ at 9 cm thickness. The decrease in radiation dosage can be attributed to the increase in Z_eff_ with the addition of BaSO_4_ to FAGP, which in turn increases the density of FAGP. Conclusions: Thus, the radiation dose can be significantly reduced with a higher ratio of BaSO_4_ to FAGP. This study shows that FAGP combined with BaSO_4_ is a promising radiation shielding material, as well as a viable alternative to OPC.

## 1. Introduction

Radiation shielding involves placing a barrier between radiation sources and surrounding materials in order to decrease radiation to a level that is safe for humans [1,2,3]. The form and thickness of the barrier are dependent on the type of radiation and amount of radiation energy. The shielding of ionizing radiation plays an important role in decreasing the dose that medical personnel are exposed to [4]. Presently, the application of radiation shields is hindered by the rarest selection of materials, which are often hard to use and difficult to install and eliminate. For X-ray radiation protection applications, shields are prepared from materials with high atomic numbers and densities such as iron, lead, and tungsten [5,6,7]. Nonetheless, barite is used in some places, such as X-ray rooms, for protection. Among conventional protective materials, lead is shown to be the most effective [8]. Since the advent of X-rays and radioactivity, versatile lead-based radiation shields have been extensively used in radiology departments across the world. Lead is the most widely used shield material for radiation protection. In this case, lead equivalents are applied as the criteria for selecting protective supplies in medical X-ray generators. Lead-based protective materials are also used by clinical personnel during X-ray image-guided interventional radiology procedures. Moreover, from an economic perspective, there is no commercial product with a similar excellent shielding capacity and workability as lead [9].

On the other hand, cement mortar prepared from ordinary Portland cement (OPC) is also used as a radiation shield because of its density and low porosity. However, the CO_2_ emissions produced during the production of ordinary Portland cement (OPC) are considerable, as one tonne of CO_2_ generated from the production of ten tonnes of OPC is emitted into the atmosphere [10,11]. Gartner (2004) reported that the manufacture of OPC requires a high inherent energy and contributes 4–8% to the total global CO_2_ production or an estimated 1.5 tons annually. In addition, the production of OPC involves the burning of vast quantities of fuel and the decomposition of limestone, which also results in significant CO_2_ emissions [12]. Therefore, a minor decrease in OPC production could drastically reduce CO_2_ emissions [13]. Geopolymer technology is reported to have the potential to reduce CO_2_ emissions by 80% [14,15]. Moreover, geopolymers are also confirmed to have a relatively stronger bond than OPC concrete [16,17]. In addition, the low shrinkage and high compressive strength of geopolymers make them a good repair material with a greater abrasion resistance compared to OPC. Geopolymers are manufactured from source materials consisting of silicon (Si) and aluminium (Al) content such as fly ash, and the solid residue obtained from coal-burning thermal power stations [18]. Geopolymers have the following properties: resistance to fire, resistance to chemical erosion, high mechanical strength (Fe), and superb solidity [19,20,21,22].

Many factors influence the solidity of geopolymers, their power and their resistance to hard environments, such as erosion performance, permeability, chemical abrasion resistance, dry retraction, resistance to the carbonization, and other factors. Previous studies mentioned that geopolymer concrete and OPC concrete have an excellent durability. Aside from this, the decrease in dry shrinkage is effective for enhancing the solidity of geopolymers [19,23,24].

Fly ash geopolymers (FAGP) are non-toxic and eco-friendly materials that produce no greenhouse gas emissions during polymerization [25]. In recent times, fly ash geopolymers have emerged as a viable alternative to OPC in the manufacture of construction and building materials [26,27,28]. FAGP have been utilized for the production of geopolymer pastes [29] and mortars [30]. In contrast to OPC, the production of FAGP does not require high amounts of energy since FA is already a by-product. Moreover, FAGP (type class F) have low calcium content, resulting in a reaction product that is inexpensive [31]. In addition, geopolymers possess exceptional mechanical properties and fire resistance [32]. A low thermal conductivity is present at high temperatures [33] and does not emit toxic fumes when subjected to heat [34]. 

The process of producing FAGP from coal ash involves mixing potassium hydroxide (KOH) and metakaolinite in a mechanical mixer at a specific ratio for 10 min. Afterwards, sodium silicate solution is added to the mixture, which is then mixed for a further 10 min. The mixture is subsequently cast in 25 mm cube moulds and vibrated for 5 min in order to release bubbles. The moulds are then sealed and kept in a sample-drying oven set at 60 °C for 1 to 7 days. After removal from the moulds, the samples are stored at room temperature for an additional 24–156 h, before performing measurements [35].

Several studies investigated the use of an OPC shield coated with BaSO_4_ as an X-ray transmission barrier, under different thicknesses and compositions [36]. The measurements were recorded at different applied voltages, ranging from 50 kVp to 120 kVp. The transmitted and backscattered X-ray was measured using an ion chamber. The results showed that the cement shield coated with BaSO_4_ could effectively absorb incident X-ray up to approximately 95% and had the ability to reduce backscattered X-ray radiation up to about 75% [36]. This present study focuses on the use of FAGP as viable substitutes for OPC in radiation shielding. BaSO_4_ was added to FAGP to enhance their shielding properties. This study also investigates the effect of BaSO_4_ concentration and sample thickness on the radiation shielding capability of the fabricated FAGP. 

The goal of this study is to prepare and evaluate the new radiation shielding capacity of fly ash geopolymer (FAGP) as a viable alternative to conventionally used ordinary Portland cement (OPC) due to the high demand for an environmentally friendly, cost-effective and non-toxic shield material.

## 2. Materials and Methods

### 2.1. OPC Preparation

**Figure 1:**
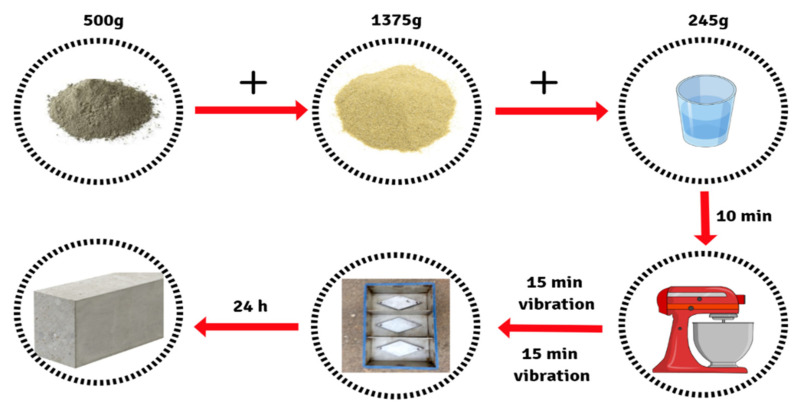
An illustration of the preparation materials and steps used for OPC preparation, which include: OPC, sand, water, mixing machine, steel moulds and prepared OPC samples, respectively.

As illustrated in Figure 1, the OPC was prepared by adding 500 g of OPC to 1375 g of sand (ratio of 2.75) [37] in a mixing machine. These ratios were selected because they produced a homogenous mixture with no settling or float of the powder materials. Afterward, 245 g of water was poured into the mixture (water/OPC ratio of 0.49) [37]. The mixture was then subjected to 10 min of machine mixing to obtain a fresh mix of OPC. The OPC mixture was subsequently poured into standard steel moulds of specific dimensions ((5 × 5 × 5) cm^3^), vibrated for 15 s using a vibrating table, and stored at room temperature for 24 h.

### 2.2. FAGP Preparation

FAPG samples were prepared by mixing sand and FA at a ratio of 1.5:1, respectively [38]. After a thorough mixing of these materials, Na_2_SiO_3_ and NaOH were added. Materials were continuously mixed until the FAGP batch was obtained [39]. The mixture was then poured into the steel moulds and vibrated for 10 s. The mould samples were subsequently wrapped with vinyl sheets to avoid moisture loss and stored for 24 h at room temperature. The samples were then placed inside an oven set at 60–70 °C for 24 h. The samples were removed and allowed to cool down to 22–25 °C for 28 days. An Excel datasheet developed at the Civil Engineering Department, School of Materials, USM, was used to calculate specific preparation ratios of FAGP. Ratios of liquid alkaline/FA, sodium silicate/NaOH, sand/FA, and water were inserted into the datasheet to automatically obtain the weights of the FAGP components (FA, sand, Na_2_SiO_3_, NaOH pellet, water and added water). The weights were found to be: 859 g of FA, 1290 g of sand, 40 g of NaOH, 275 g of Na_2_SiO_3_, 90 g of H_2_O and 60 g of added water. After fabrication, the samples were subjected to EDX measurements to analyse the elemental percentages, which were then used in the Z_eff_ calculation.

### 2.3. Leaching Text on Synthesized Samples

#### X-ray Dose Measurement Setup

**Figure 2:**
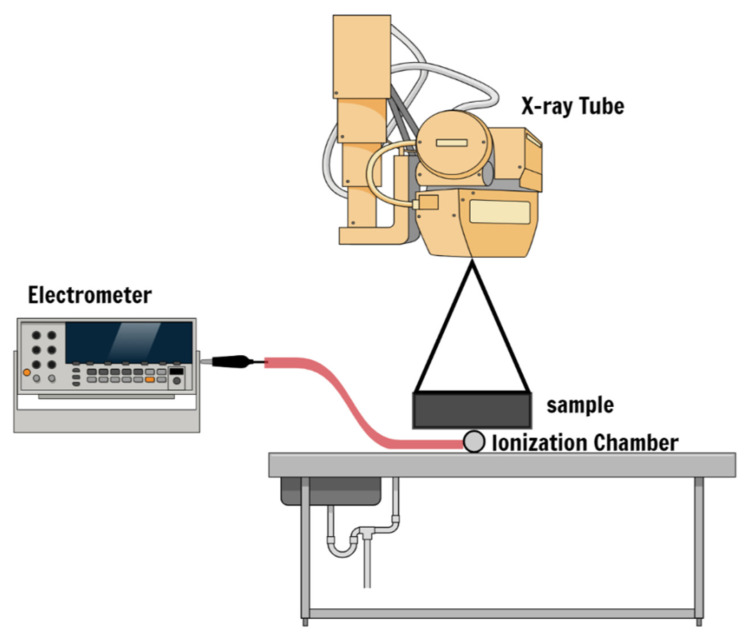
Schematic diagram illustrating the general set-up.

Five samples (OPC and FAGP with 0, 5, 10 and 15% of BaSO_4_) with different thicknesses (3, 6 and 9 cm) were irradiated using a Toshiba X-ray machine, as shown in Figure 2. A 1 cm^3^ ionization chamber type 7734 (PTW, Freiburg) was connected to a SUPERMAX electrometer and irradiated to measure the air kerma. The absorbed dose was calculated using Equation (1) [40]:

(1) $$D_{\mathrm{air}}=\frac{Q}{m_{\mathrm{air}}}\left[\frac{W_{\mathrm{air}}}{e}\right]$$

where Q symbolizes liberated ion charges, m_air_ is the mass of air related to absorbed dose, W_air_/e denotes the mean energy required to produce an ion pair in air per unit charge (the current value for dry air is 33.97 eV/ion pair or 33.97 J/C), with ρ_air_ = 1.25 × 10^−3^ g/cm^3^, v_air_ = 3.46 cm^3^, m_air_ = ρv_air_ = 4.33 × 10^−6^ kg. 

## 3. Results

### 3.1. EDX and Effective Atomic Number (Z_eff_)

An energy-dispersive X-ray (EDX) was used to analyse the elemental composition of the samples, which included OPC, FAGP and FAGP incorporated with 5%, 10% and 15% of BaSO_4_. The elemental percentages of the samples are outlined in Table 1.

Table 1 shows various elemental ratios in OPC and fly ash geopolymers (FAGP) material prepared without and with (5, 10, and 15 % of BaSO_4_). However, in the table, the frequent use of BaSO_4_ can be observed because the modulation of BaSO_4_ increases the density of the FAGP material, allowing increasing its solidity more than the cement material.

**Figure 3:**
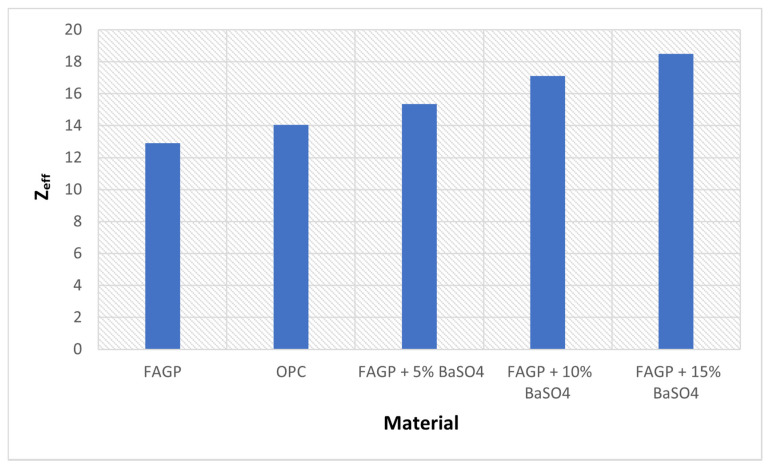
Histogram plot of Z_eff_ values for OPC and FAGP with different concentration of BaSO_4_.

However, Figure 3 shows that the Z_eff_ of OPC (14.04) is higher than that of FAGP (12.9). Moreover, the results indicated that the Zeff for the addition of BaSO_4_ particles achieved a high Z_eff_ value, and this increased the material solidity.

### 3.2. Effect of Thickness on the Radiation Shielding Capacities of OPC and FAGP

The transmission of radiation energy through OPC and FAGP (incorporated with 0, 5, 10 and 15% BaSO_4_) prepared under different thicknesses (3, 6 and 9 cm) was examined. The OPC material showed comparably better radiation shielding than FAGP without BaSO_4_ for all thicknesses.

## 4. Discussion

It can be deduced from the EDX of the FAGP samples (combined with different concentrations of BaSO_4_) that the percentage of oxygen in cement mortar exceeds that of the FAGP. In contrast, the percentages of Si and Al are lower in cement mortar (OPC) compared to the FAGP. It important to note that the inclusion of BaSO_4_ increases the density of the FAGP material, making it denser than the cement material. However, the addition of BaSO_4_ did not significantly affect the elemental composition of the FAGP. Nonetheless, the density effect of BaSO_4_ is significant because of the strong relationship between the effective atomic number and attenuation coefficient. The effective atomic number of a material (Z_eff_) is the atomic number of a hypothetical element that attenuates photons at the same rate as the material. Z_eff_ can be calculated using Equation (2):

(2) $$Z_{\mathrm{eff}}={\left[a_{1}Z_{1}^{2.94}+a_{2}Z_{2}^{2.94}+\ldots +a_{n}Z_{n}^{2.94}\right]}^{\frac{1}{2.94}}$$

In Equation (2), the Z_eff_ of FAGP incorporated with 5% of BaSO_4_ was found to be 18.5. The Z_eff_ values increased with the addition of BaSO_4_, which could be attributed to the weight (%) of barium (Ba). 

After the addition of different percentages of BaSO_4_ to FAGP, Z_eff_ increased to 14.6 and 18.5 for BaSO_4_ ratios of 5 and 15%, respectively. As the Z_eff_ of the material significantly increased with the additional weight (%) of incorporated BaSO_4_, the density of the FAGP improved from 2.13 g/cm^3^ with the inclusion of 10% BaSO_4_ to 2.21 g/cm^3^ with 15% BaSO_4_. Thus, the increase in Z_eff_ was due to the high density of BaSO_4_ (4.5 g /cm^3^). This result supports the use of FAGP as a viable radiation shielding material in lieu of OPC. 

**Figure 4:**
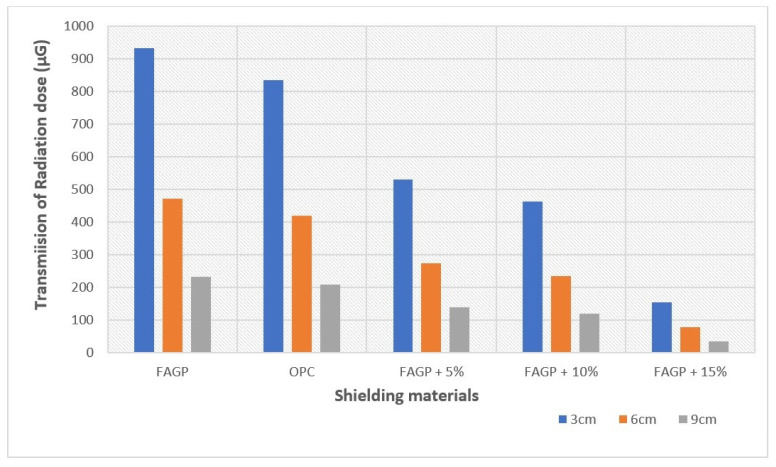
A diagram showing the differential dose absorption through OPC and different thicknesses of FAGP.

As observed in Figure 4, the radiation transmission decreased as the shield thickness increased, but FAGP combined with BaSO_4_ achieved a better reduction in radiation dose with increase in BaSO_4_ ratio from 0 to 15%. The transmission of radiation energy decreased from 932 to 154.23 µGy at 3 cm thickness, from 470.72 to 78.45 µGy at 6 cm thickness, and from 232.40 to 34.68 µGy at 9 cm thickness, with FAGP combined with a BaSO_4_ ratio of 15% showing the least dosage of transmitted radiation. The decrease in radiation dosage can be attributed to the increase in Z_eff_ with the addition of BaSO_4_ to FAGP, which in turn increases the density of FAGP.

This study evaluated the radiation shielding capacity of FAGP incorporated with BaSO_4_ as a more viable alternative to OPC. Compared to other previous studies [41], using FAGP incorporated with BaSO_4_ provided an effective shielding of X-rays that were required in medical, aviation and nuclear fields. Therefore, a novel X-ray-shielding FAGP/BaSO_4_ mixing sand and FA were designed. 

## 5. Conclusions

This study investigated the effect of BaSO_4_ concentration (0, 5, 10, and 15%) and sample thickness (3, 6, 9 cm) on the radiation shielding capability of the fabricated FAGP. The lowest dosage of transmitted radiation (34.68 µGy) and highest effective atomic number (Z_eff_) were achieved with FAGP combined with 15% BaSO_4_ at 9 cm thickness. It is inferred that the radiation dose can be significantly decreased by increasing the concentration of BaSO_4_ in FAGP. However, OPC is a more effective radiation shielding material compared to FAGP in the absence of BaSO_4_. The positive impact of BaSO_4_ is due to its intrinsically high density. This study concludes that FAGP combined with BaSO_4_ is a promising radiation shielding material, as well as a potential alternative to OPC. Moreover, FAGP is environmentally friendly, cost-effective, and non-toxic.

## Figures and Tables

**Table 1:** Different elemental ratios in OPC, fly ash geopolymer material (FAGP) prepared without and with (5, 10, and 15 % of BaSO_4_).

Element	OPC W (%)	FAGP W (%)	FAGP + 5% BaSO_4_ W (%)	FAGP + 10% BaSO_4_ W (%)	FAGP + 15% BaSO_4_ W (%)
**C**	12.94	10.98	11.19	10.82	10.11
**O**	56.47	44.04	47.18	46.01	45.47
**Na**		14.47	13.54	14.03	14.53
**Mg**	11.41	1.67	0.22	0.22	0.21
**Al**	0.23	6.51	7.78	7.22	6.55
**Si**	1.11	10.2	7.64	8.61	9.55
**P**		0.37	0.19	0.11	0.17
**S**	0.32	0.4	0.25	0.71	1.22
**K**		0.07	0.12	0.37	0.56
**Ca**	17.52	8.95	7.04	6.34	5.85
**Ti**		0.29	0.94	0.83	0.75
**Fe**		2.05	2.8	2.13	1.6
**Ba**			1.11	2.25	3.43
**Total**	100	100	100	100	100

## Data Availability

Data has been included in the manuscript.

## References

[1] Kumar A. (2017). Gamma ray shielding properties of PbO-Li_2_O-B_2_O_3_ glasses. Radiat. Phys. Chem..

[2] Basu P., Sarangapani R., Venkatraman B. (2019). Gamma ray buildup factors for conventional shielding materials and buildup factors computed for tungsten with a thickness beyond 40 mean free paths. Appl. Radiat. Isot..

[3] Oglat A.A. (2020). Acceptance experimentation and quality monitor of X-ray radiography units. Radiat. Phys. Chem..

[4] Kalita J., Chithambo M. (2019). Thermoluminescence and infrared light stimulated luminescence of limestone (CaCO_3_) and its dosimetric features. Appl. Radiat. Isot..

[5] Dong M., El-Mallawany R., Sayyed M., Tekin H. (2017). Shielding properties of 80TeO_2_–5TiO_2_–(15 − x) WO_3–x_AnOm glasses using WinXCom and MCNP5 code. Radiat. Phys. Chem..

[6] Kaur K., Singh K., Anand V. (2016). Structural properties of Bi_2_O_3_–B_2_O_3_–SiO_2_–Na_2_O glasses for gamma ray shielding applications. Radiat. Phys. Chem..

[7] Oglat A.A. (2020). Chemistry. Studying the radiation absorption and scattering of gamma rays by using different absorbers. Radiat. Phys. Chem..

[8] Tashlykov O., Shcheklein S.Y., Lukyanenko V.Y., Mikhaylova A., Russkikh I., Seleznev Y.N., Kozlov A. (2016). The optimization of radiation protection composition. Nucl. Energy Technol..

[9] Boeykens S., Redondo N., Obeso R.A., Caracciolo N., Vázquez C. (2019). Chromium and Lead adsorption by avocado seed biomass study through the use of Total Reflection X-ray Fluorescence analysis. Appl. Radiat. Isot..

[10] Davidovits J. (1994). High-alkali cements for 21st century concretes. Spec. Publ..

[11] Rehan R., Nehdi M. (2005). Carbon dioxide emissions and climate change: Policy implications for the cement industry. Environ. Sci. Policy.

[12] Kong D.L., Sanjayan J.G. (2008). Damage behavior of geopolymer composites exposed to elevated temperatures. Cem. Concr. Compos..

[13] Gunasekara M., Law D., Setunge S. Effect of composition of fly ash on compressive strength of fly ash based geopolymer mortar. Proceedings of the 23rd Australasian Conference on the Mechanics of Structures and Materials.

[14] Davidovits J. (1994). Global warming impact on the cement and aggregates industries. World Resour. Rev..

[15] BrahimiMoussa S., Benamar M.E.A., LounisMokrani Z. (2019). Characterization of the chemical and structural modifications induced by gamma rays on the MAGIC polymer gel. Radiat. Phys. Chem..

[16] Hu S., Wang H., Zhang G., Ding Q. (2008). Bonding and abrasion resistance of geopolymeric repair material made with steel slag. Cem. Concr. Compos..

[17] Avcıbaşı U., Ateş B., Ünak P., Gümüşer F.G., Gülcemal S., Ol K.K., Akgöl S., Tekin V. (2019). A novel radiolabeled graft polymer: Investigation of the radiopharmaceutical potential using Albino Wistar rats. Appl. Radiat. Isot..

[18] Hardjito D., Wallah S.E., Sumajouw D.M., Rangan B.V. (2004). On the development of fly ash-based geopolymer concrete. ACI Mater. J.-Am. Concr. Inst..

[19] Cong P., Cheng Y., Engineering T. (2021). Advances in geopolymer materials: A comprehensive review. J. Traffic Transp. Eng..

[20] Kaze C.R., Lecomte-Nana G.L., Kamseu E., Camacho P.S., Provis J.L., Duttine M., Wattiaux A., Melo U.C., Research C. (2021). Mechanical and physical properties of inorganic polymer cement made of iron-rich laterite and lateritic clay: A comparative study. Cem. Concr. Res..

[21] Kaze C.R., Lecomte-Nana G.L., Adesina A., Nemaleu J.G.D., Kamseu E., Melo U.C., Composites C. (2022). Influence of mineralogy and activator type on the rheology behaviour and setting time of laterite based geopolymer paste. Cem. Concr. Res..

[22] Hu Y., Liang S., Yang J., Chen Y., Ye N., Ke Y., Tao S., Xiao K., Hu J., Hou H. (2019). Role of Fe species in geopolymer synthesized from alkali-thermal pretreated Fe-rich Bayer red mud. Constr. Build. Mater..

[23] Yang X., Yan Z., Yin S., Gao Q., Li W. (2022). The Ratio Optimization and Strength Mechanism of Composite Cementitious Material with Low-Quality Fly Ash. Gels.

[24] Mirković M., Kljajević L., Dolenec S., Nenadović M., Pavlović V., Rajačić M., Nenadović S.J.G. (2021). Potential Usage of Hybrid Polymers Binders Based on Fly Ash with the Addition of PVA with Satisfying Mechanical and Radiological Properties. Gels.

[25] Hardjito D., Rangan B.V. (2005). Development and Properties of Low-Calcium Fly Ash-Based Geopolymer Concrete.

[26] Palomo A., Grutzeck M., Blanco M. (1999). Alkali-activated fly ashes: A cement for the future. Cem. Concr. Res..

[27] Hardjito D. (2005). Studies of Fly Ash-Based Geopolymer Concrete.

[28] Nawy E.G. (2008). Concrete Construction Engineering Handbook.

[29] Temuujin J., Van Riessen A., Williams R. (2009). Influence of calcium compounds on the mechanical properties of fly ash geopolymer pastes. J. Hazard. Mater..

[30] Lee W., Van Deventer J. (2004). The interface between natural siliceous aggregates and geopolymers. Cem. Concr. Res..

[31] Wallah S., Rangan B.V. (2006). Low-Calcium Fly Ash-Based Geopolymer Concrete: Long-Term Properties.

[32] Palomo A., Macias A., Blanco M., Puertas F. Physical, chemical and mechanical characterization of geopolymers. Proceedings of the 9th International Congress on the Chemistry of Cement.

[33] Luna-Galiano Y., Cornejo A., Leiva C., Vilches L., Fernández-Pereira C. (2015). Properties of fly ash and metakaolín based geopolymer panels under fire resistance tests. Mater. Construc..

[34] Duxson P., Lukey G.C., van Deventer J.S. (2006). Thermal evolution of metakaolin geopolymers: Part 1–Physical evolution. J. Non-Cryst. Solids.

[35] Wang J., Cheng T. Production geopolymer materials by coal fly ash. Proceedings of the 7th International Symposium on East Asian Resources Recycling Technology.

[36] Naji A.T., Jaafar M.S., Ali E. (2015). X-ray Protection Using Mixture of Cement Shielding with Barium Sulfate. J. Sci. Technol..

[37] (1993). Standard Test Method for Compressive Strength of Hydraulic Cement Mortars.

[38] Mijarsh M.J.A., Megat Johari M.A., Ahmad Z.A. (2014). Synthesis of geopolymer from large amounts of treated palm oil fuel ash: Application of the Taguchi method in investigating the main parameters affecting compressive strength. Constr. Build. Mater..

[39] Granizo M.L., Alonso S., Blanco-Varela M.T., Palomo A. (2002). Alkaline activation of metakaolin: Effect of calcium hydroxide in the products of reaction. J. Am. Ceram. Soc..

[40] Oglat A.A., Alshipli M., Sayah M.A., Farhat O., Ahmad M.S., Abuelsamen A. (2021). Fabrication and characterization of epoxy resin-added Rhizophora spp. particleboards as phantom materials for computer tomography (CT) applications. J. Adhes..

[41] Jiang X., Zhu X., Chang C., Liu S., Luo X. (2019). X-ray shielding structural and properties design for the porous transparent BaSO4/cellulose nanocomposite membranes. Int. J. Biol. Macromol..

